# Optimal water concentration for aqueous Li^+^ intercalation in vanadyl phosphate†

**DOI:** 10.1039/d0sc04647g

**Published:** 2021-02-11

**Authors:** Dan Sun, Masashi Okubo, Atsuo Yamada

**Affiliations:** Department of Chemical System Engineering, School of Engineering, The University of Tokyo Hongo 7-3-1, Bunkyo-ku Tokyo 113-8656 Japan yamada@chemsys.t.u-tokyo.ac.jp; Elemental Strategy Initiative for Catalysts & Batteries (ESICB), Kyoto University Nishikyo-ku Kyoto 615-8510 Japan

## Abstract

Development of high-performance aqueous batteries is an important goal for energy sustainability owing to their environmental benignity and low fabrication costs. Although a layered vanadyl phosphate is one of the most-studied host materials for intercalation electrodes with organic electrolytes, little attention has been paid to its use in aqueous Li^+^ systems because of its excessive dissolution in water. Herein, by controlling the water concentration, we demonstrate the stable operation of a layered vanadyl phosphate electrode in an aqueous Li^+^ electrolyte. The combination of experimental analyses and density functional theory calculations reveals that reversible (de)lithiation occurs between dehydrated phases, which can only exist in an optimal water concentration.

## Abstract 2

We demonstrate aqueous Li^+^ intercalation chemistry of vanadyl phosphate in the form of monohydrates, which are only stable in an optimal water-concentration environment.

## Graphical abstract

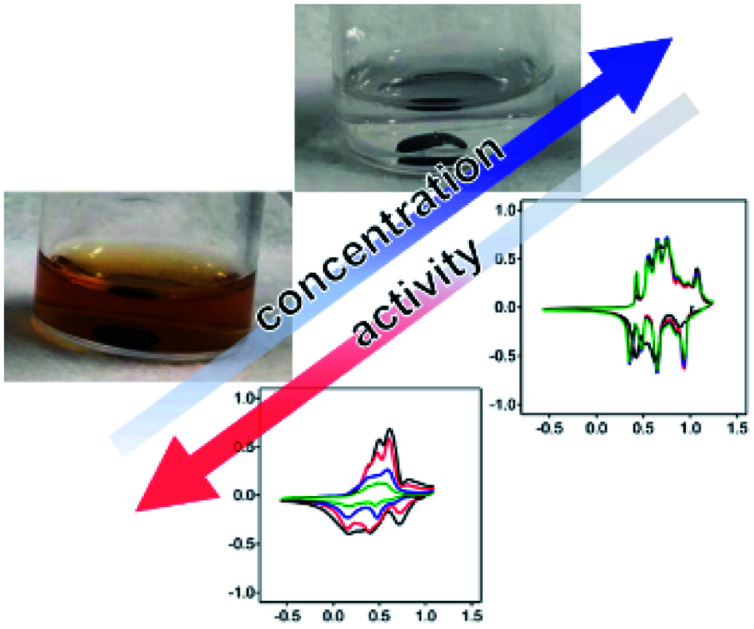

## Introduction

Lithium-ion batteries (LIBs) dominate the battery market for portable electronics and electric vehicles owing to their long lifetime, high efficiency, and high energy densities. However, LIBs are comprised of flammable and costly organic electrolytes, which unexceptionally accompany both safety hazards and high fabrication costs.^1^ Batteries that utilize aqueous electrolytes are expected to provide more operational safety, affordability, high power, and environmental benignity, all of which are favorable for large-scale stationary systems and electric vehicle operations.^2,3^ Although the intrinsically narrow electrochemical stability window for water as an electrolyte solvent (*∼*1.23 V) imposed severe limitations on any practical applications of aqueous batteries, novel strategies involving highly concentrated aqueous electrolytes have achieved much wider electrochemical potential windows (>3 V), thereby paving a path for the development of more practical aqueous batteries.^4–8^ Importantly, by exploiting a specialized solution structure without free water,^9–14^ highly concentrated aqueous electrolytes can also provide unexpected environments for electrode materials that have been considered ‘useless’ in conventional dilute aqueous electrolytes.^15^

**Fig. 1:**
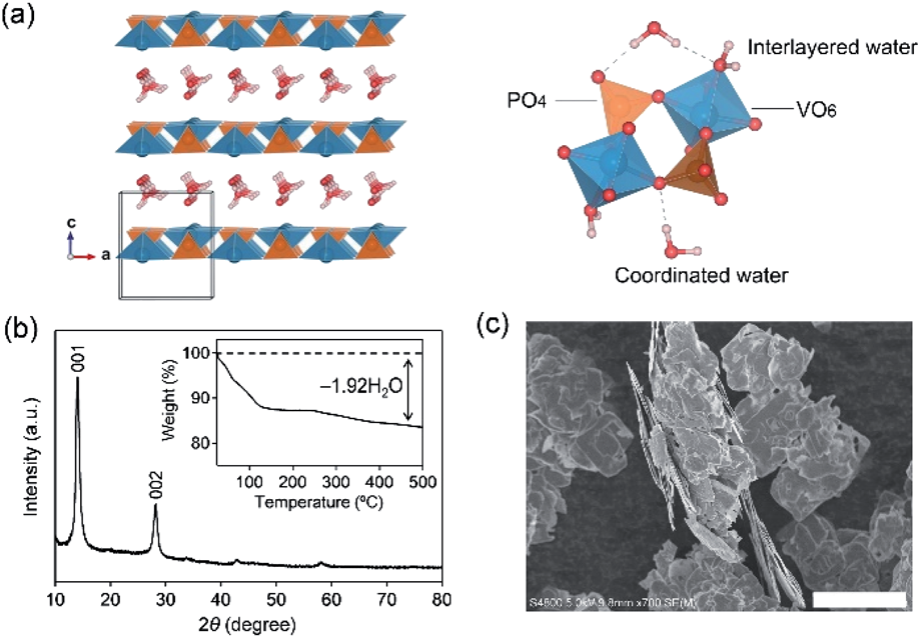
(a) Crystal structure of VOPO_4_·*n*H_2_O. V (dark cyan), P (orange), O (red), and H (gray) atoms are shown. (b) Powder X-ray diffraction pattern and (c) scanning electron microscopy image of VOPO_4_·*n*H_2_O. The inset in (b) is the thermal gravimetric analysis profile of VOPO_4_·*n*H_2_O.

The selection criteria of electrode materials for aqueous batteries are (i) suitable redox potential, (ii) durability against water, (iii) no side reactions, (iv) good reversibility, and (v) low cost. Fig. S1† lists selected electrode materials that are potentially compatible with aqueous electrolytes. Among them, for instance, hydrated vanadyl phosphate (VOPO_4_·*n*H_2_O, Fig. 1a) is a versatile layered host for intercalation chemistry, which has been ascertained using various organic electrolyte systems.^16–21^ However, except for a few very recent reports of its application to aqueous H^+^/Zn^2+^ batteries,^22,23^ VOPO_4_·*n*H_2_O has rarely been considered promising in aqueous systems, due simply to dissolution and decomposition of VOPO_4_·*n*H_2_O in aqueous electrolytes.

VOPO_4_·2H_2_O possesses a bilayer structure where a VOPO_4_ layer is composed of corner-sharing VO_6_ octahedra and PO_4_ tetrahedra, with a layer distance of 7.25 Å along the *c* axis, as illustrated in Fig. 1a. When VOPO_4_·*n*H_2_O is immersed in liquids that possess high water concentration *c*(H_2_O) (*e.g.*, pure water or dilute aqueous solutions), surface vanadium atoms are readily coordinated by the water to form soluble vanadium aquo complexes. Furthermore, high-concentration water is prone to intercalate into an interlayer space to exfoliate VOPO_4_ layers, accelerating the dissolution process. Alternatively, an extremely low-*c*(H_2_O) environment (*e.g.*, highly concentrated aqueous solutions) is expected to desorb interlayer water between VOPO_4_ layers. Since interlayer water plays an important role in both structural integrity and ion diffusion, the intercalation chemistry may be largely altered *via* the control of *c*(H_2_O) of aqueous electrolytes for achieving reversible lithium-ion (de)intercalation in VOPO_4_·*n*H_2_O. Herein, we demonstrate the stable charge/discharge operation of VOPO_4_·*n*H_2_O through accurate control of *c*(H_2_O) in aqueous Li^+^ electrolytes.

## Results and discussion

VOPO_4_·*n*H_2_O was synthesized by refluxing V_2_O_5_ and H_3_PO_4_,^24^ and the resulting powder was dried under vacuum overnight at 80 °C. The powder X-ray diffraction (XRD) pattern (Fig. 1b) corresponds to tetragonal VOPO_4_·*n*H_2_O, and a 001 diffraction at 2*θ* ≈ 14° indicates an interlayer distance of 7.35 Å. The thermogravimetric (TG) analysis (inset of Fig. 1b) reveals a water content per formula unit of *n* = 1.92. The scanning electron microscopy image (Fig. 1c) shows a lamellar morphology with a lateral size ranging from 10 to 100 μm.

**Fig. 2:**
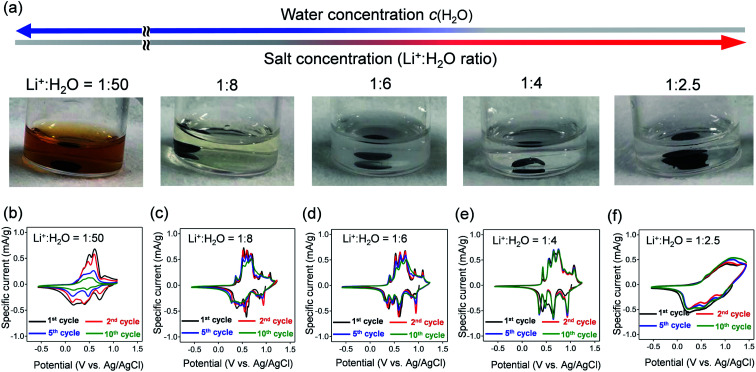
(a) Photographs of aqueous Li^+^ electrolytes after immersing VOPO_4_·*n*H_2_O for 30 days. The Li^+^ : H_2_O ratio ranges from 1 : 50 (LiTFSI/50H_2_O), 1 : 8 (LiTFSI/8H_2_O), 1 : 6 (LiTFSI/6H_2_O), 1 : 4 (LiTFSI/4H_2_O) to 1 : 2.5 (water-in-salt, LiTFSI/2.5H_2_O). (b–f) Cyclic voltammetry curves of VOPO_4_·*n*H_2_O in aqueous Li^+^ electrolytes at various Li^+^ : H_2_O ratios at a scan rate of 0.5 mV s^−1^.

The dissolution durability of VOPO_4_·*n*H_2_O was tested using aqueous Li^+^ electrolytes (Fig. 2a). After immersing VOPO_4_·*n*H_2_O in aqueous electrolytes at various Li^+^ : H_2_O ratios for 30 days, the color of the dilute electrolyte (Li^+^ : H_2_O = 1 : 50, high *c*(H_2_O)) changed to orange, while concentrated electrolytes (*e.g.*, Li^+^ : H_2_O = 1 : 4, low *c*(H_2_O)) remained transparent, indicating effective suppression of vanadium ion dissolution by lowering *c*(H_2_O).

Cyclic voltammetry (CV) was conducted to evaluate the electrochemical properties of VOPO_4_·*n*H_2_O under various *c*(H_2_O) conditions (Fig. 2b–f). The CV curves for the dilute aqueous electrolyte (Li^+^ : H_2_O = 1 : 50, high *c*(H_2_O)) (Fig. 2b) show severe decay of current flow with repeating CV cycles. The decay of current flow coincides with the change in the electrolyte color to orange, arising from the dissolution of vanadium in a high-*c*(H_2_O) environment. In contrast, upon increasing the salt concentration (lowering *c*(H_2_O)) to a Li^+^ : H_2_O ratio of 1 : 4, intense multiple current flows gradually emerge with minimal polarization, indicating reversible (de)lithiation without parasitic reactions/dissolution. However, when further lowering *c*(H_2_O) to a Li^+^ : H_2_O ratio of 1 : 2.5, the cyclic voltammetry (CV) curve shows broad cathodic/anodic current flows with large polarization (Fig. 2f), which indicates sluggish (de)lithiation of VOPO_4_·*n*H_2_O in an environment of overly low *c*(H_2_O). Electrochemical impedance spectroscopy (Fig. S2†) shows the specific increase of both series resistance and charge-transfer resistance specifically when the Li^+^ : H_2_O ratio changes from 1 : 4 to 1 : 2.5. Presumably, the low ionic conductivity of the highly concentrated aqueous electrolyte increases the series resistance while the strong coulombic attraction between Li^+^ and TFSI^−^ (contact-ion pair) increases the charge-transfer resistance, both of which cause the sluggish (de)lithiation of VOPO_4_·*n*H_2_O.^14^ Therefore, an optimal-*c*(H_2_O) environment (Li^+^ : H_2_O = 1 : 4) is required for reversible (de)lithiation of VOPO_4_·*n*H_2_O in aqueous systems.

**Fig. 3:**
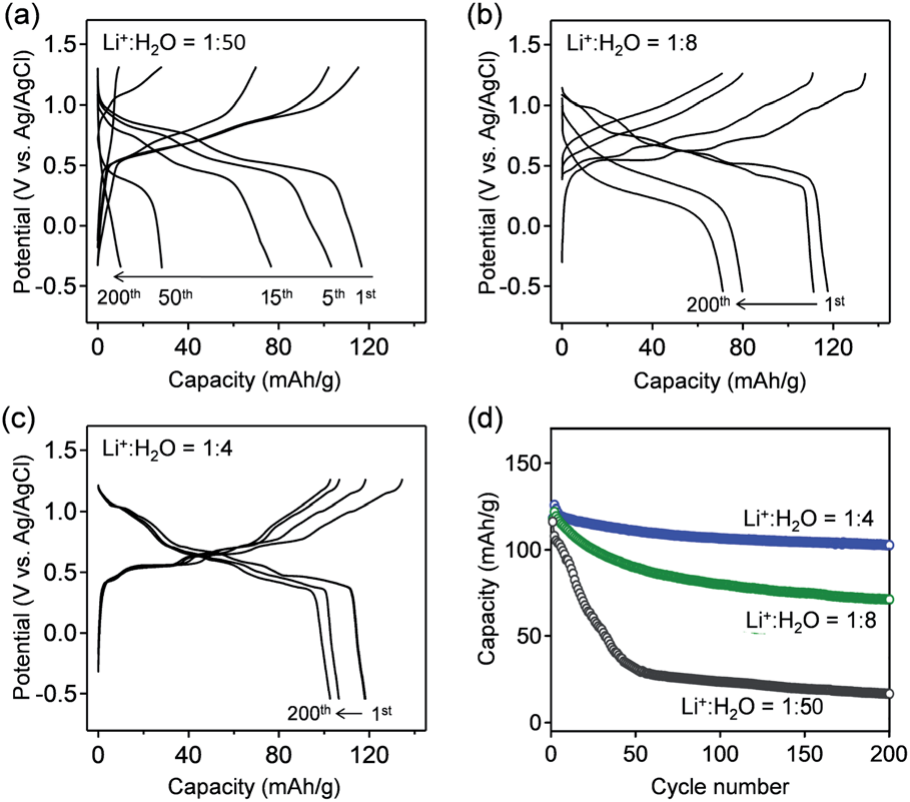
Charge/discharge curves of VOPO_4_·H_2_O electrodes with aqueous Li^+^ electrolytes. The Li^+^ : H_2_O ratios are (a) 1 : 50 (LiTFSI/50H_2_O), (b) 1 : 8 (LiTFSI/8H_2_O), and (c) 1 : 4 (LiTFSI/4H_2_O), respectively. The specific current is 1 A g^−1^. (d) Capacity retention during 200 charge/discharge cycles.

Fig. 3a–c show the galvanostatic charge/discharge curves of VOPO_4_·*n*H_2_O in various *c*(H_2_O) environments at a specific current of 1 A g^−1^. The delivered capacity with a high-*c*(H_2_O) aqueous electrolyte (Fig. 3a) drastically decreases upon cycling, while the potential polarization increases, presumably because of the vanadium dissolution. Upon gradually lowering *c*(H_2_O) to an optimal range (Li^+^ : H_2_O = 1 : 4), the potential profiles exhibit distinct multiple plateaus without substantial polarization, indicating highly reversible (de)lithiation (Fig. 3b and c). However, when further lowering *c*(H_2_O) to Li^+^ : H_2_O = 1 : 2.5, the polarization between lithiation/delithiation significantly increases to reduce the reversible capacity to approximately 85 mA h g^−1^ (Fig. S3†). These results are consistent with the CV results (Fig. 2). The reversible capacity of 118 mA h g^−1^ in the first cycle (Li^+^ : H_2_O = 1 : 4) corresponds to 0.8 Li^+^ intercalation per formula unit. The reversible reduction/oxidation of vanadium (V^5+^/V^4+^) upon charge/discharge, which was confirmed by V K-edge X-ray absorption spectroscopy (Fig. S4†), supports the occurrence of reversible (de)lithiation of VOPO_4_·*n*H_2_O. Furthermore, owing to the optimal-*c*(H_2_O) environment, 80% of the initial capacity is retained after 200 cycles (Fig. 3d), which is much higher than that for a high-*c*(H_2_O) aqueous electrolyte (7%).

**Fig. 4:**
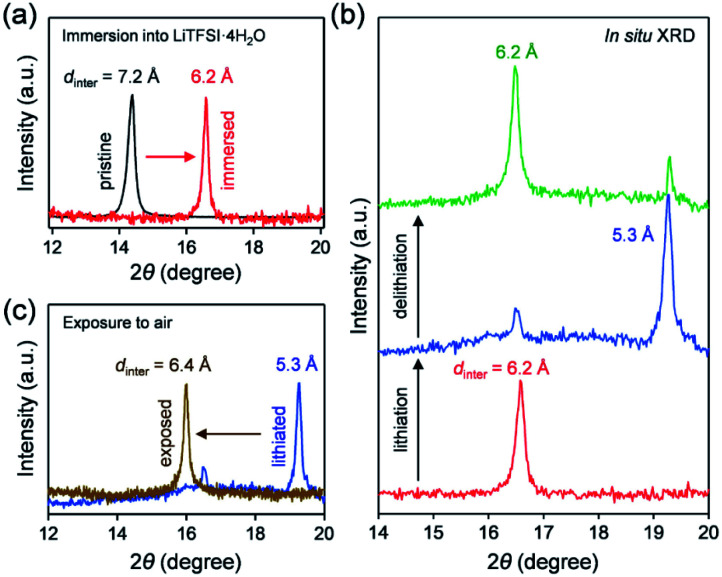
Structural changes of VOPO_4_·*n*H_2_O in LiTFSI/4H_2_O. The 001 diffraction peak (a) before and after immersion in LiTFSI/4H_2_O, (b) after lithiation and delithiation in LiTFSI/4H_2_O, and (c) before and after exposure of a lithiated sample to the ambient atmosphere. *d*_inter_ denotes the interlayer distance.

To clarify the phase diagram of Li_*x*_VOPO_4_·*n*H_2_O in the optimal *c*(H_2_O) environment, *in situ* XRD experiments were conducted (Fig. 4a–c). Before immersion in the optimal-*c*(H_2_O) electrolyte, VOPO_4_·*n*H_2_O in a composite electrode possesses an interlayer distance of 7.2 Å. The calculated interlayer distance of VOPO_4_·2H_2_O (7.25 Å, Fig. S5 and Table S1†) agrees with the experimental value, and the water content of VOPO_4_·*n*H_2_O in a pristine electrode is estimated as *n* ≈ 2, which is in good agreement with the TG result (the inset of Fig. 1b). However, the *in situ* XRD pattern for VOPO_4_·*n*H_2_O immersed in a LiTFSI/4H_2_O electrolyte shows the shift of 001 diffraction to a higher diffraction angle (Fig. 4a), indicating the decrease of the interlayer distance to 6.2 Å. This value is consistent with the calculated interlayer distance for VOPO_4_·H_2_O (6.16 Å, Fig. S5 and Table S1†). Therefore, the low-*c*(H_2_O) environment dehydrates VOPO_4_·*n*H_2_O to a monohydrate phase (*n* = 1). Upon lithiation/delithiation, a new 001 diffraction, corresponding to an interlayer distance of 5.3 Å, emerges/diminishes at the expense of the original 001 diffraction (Fig. 4b), suggesting biphasic (de)lithiation. It is noteworthy that the lithiated/delithiated monohydrate phases are stable only in the optimal-*c*(H_2_O) environment; for instance, the lithiated phase immediately becomes hydrated to a dihydrate phase (*n* = 2) after being exposed to the ambient atmosphere (Fig. 4c).

**Fig. 5:**
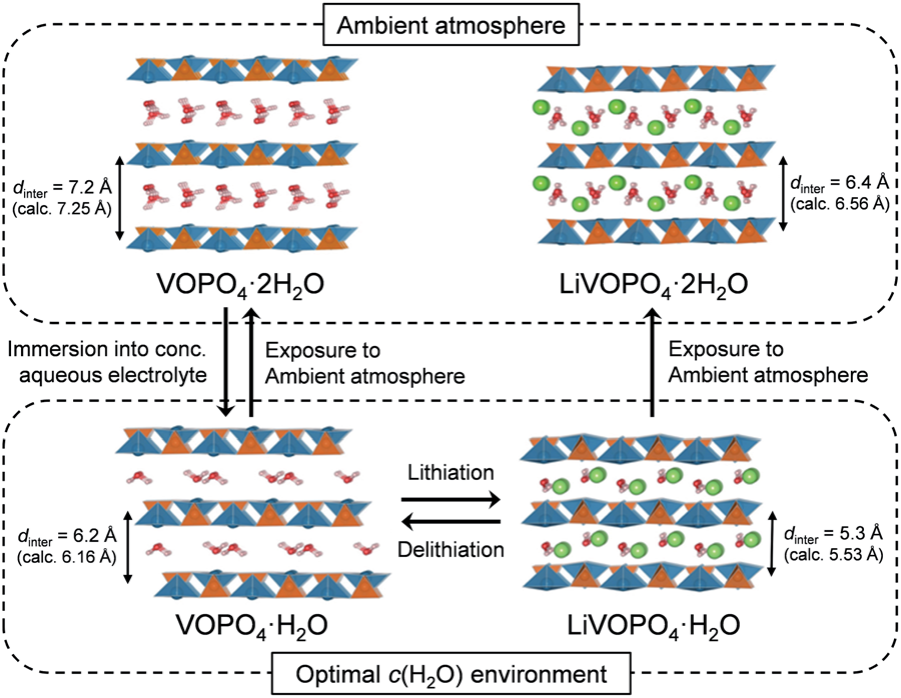
Schematic illustration of the phase diagram of VOPO_4_·*n*H_2_O in the optimal-*c*(H_2_O) environment.

A phase diagram of Li_*x*_VOPO_4_·*n*H_2_O in the optimal-*c*(H_2_O) environment is postulated based on the experimental and simulation results as schematized in Fig. 5. The equilibrium between interlayer water in VOPO_4_·*n*H_2_O and low-concentration water in concentrated aqueous electrolytes drives dehydration of VOPO_4_·*n*H_2_O to the monohydrate phase (*n* = 1). Although exhibiting reversible Li^+^ (de)intercalation, the monohydrate phases can only be stabilized in a low-*c*(H_2_O) environment, and exposure to the ambient atmosphere immediately causes hydration to the dihydrate phase (*n* = 2).

## Conclusions

In summary, the stable operation of VOPO_4_·*n*H_2_O electrodes in an optimal-*c*(H_2_O) environment was successfully demonstrated. The reversible (de)lithiation occurs specifically in monohydrate phases, which are only stable in the optimal-*c*(H_2_O) environment. This study provides novel insights into the reaction mechanism and phase diagram of VOPO_4_·*n*H_2_O in a low-*c*(H_2_O) environment, and more importantly points to research opportunities to enrich aqueous ion intercalation chemistry by controlling water concentration.

## Methods

VOPO_4_·*n*H_2_O was synthesized *via* a reflux method: V_2_O_5_ (6 g), H_2_O (144 mL) and H_3_PO_4_ (82%, 57.75 g) at 125 °C for 16 h. After cooling to room temperature, the resulting yellow precipitate was collected by centrifugation and washed three times with water and acetone. Thereafter, the powder was dried under vacuum overnight at 80 °C.

X-ray diffraction patterns were recorded on a Bruker AXS D8 Advance X-ray diffractometer using Co Kα radiation. Microstructure analyses were performed using a scanning electron microscope (Hitachi, S-4800) at a beam accelerating voltage of 5 kV. The oxidation states of the samples were measured by V K-edge XANES at the beamline 8B of Photon Factory (PF), High Energy Accelerator Research Organization (KEK) Tsukuba, Japan. The crystal water content of the as-synthesized VOPO_4_·*n*H_2_O sample was confirmed by TG (NETZSCH, STA2500).

VOPO_4_·*n*H_2_O electrodes were fabricated by grinding a mixture containing VOPO_4_·*n*H_2_O, carbon black (super P) and polytetrafluoroethylene (PTFE) in a weight ratio of 75 : 10 : 15 for 20 min, and then it was rolled into an electrode film using a rolling machine with a fixed gap of 250 μm. For the preparation of aqueous electrolytes, lithium bis-(trifluoromethanesulfonyl)imide (LiTFSI) was dissolved in ultrapure water as LiTFSI·*n*H_2_O (*n* = 2.5, 4, 6, 8, and 50). The electrochemical performance of VOPO_4_·*n*H_2_O electrodes was evaluated using a three-electrode system (Ag/AgCl and active carbon as the reference and counter electrodes, respectively). CR2032-type coin cells were assembled to measure the galvanostatic charge/discharge properties of the electrodes. The cell consists of a VOPO_4_·*n*H_2_O electrode, active carbon anode and glass fiber separator (GF/F, Whatman). Electrochemical impedance spectroscopy (VMP3 potentiostat, Biologic) was performed at the open circuit potential with an amplitude of 10 mV in the frequency range of 10 mHz to 200 kHz.

First-principles calculations were performed using the Vienna *Ab initio* Simulation Package (VASP),^25^ based on density functional theory (DFT).^26,27^ The exchange–correlation energy is calculated using general gradient approximation (GGA) with the Perdue–Burke–Ernzerhof (PBE) exchange–correlation functional.^28^ Furthermore, in our calculations, Hubbard *U* corrections (GGA + *U*) were adopted with *U* − *J* = 3.1 for vanadium. The effect of van der Waals interactions was estimated and implemented in the optimized exchange van der Waals functional B86b of the Becke (optB86b vdW) functional.^29,30^ The plane wave cutoff energy was 580 eV. The convergence condition for the energy is 10^−4^ eV, and the structures were relaxed until the force on each atom was less than 0.01 eV Å^−1^. Spin polarization was considered in all calculations. The *k*-point meshes of 7 × 7 × 7 and 14 × 14 × 14 in the Monkhorst–Pack sampling scheme were used for geometry optimization and electronic self-consistent computation, respectively.^31^

## Conflicts of interest

There are no conflicts to declare.

## Supplementary Material

SC-012-D0SC04647G-s001
