# Supplementary material for: Optimal water concentration for aqueous Li+ intercalation in vanadyl phosphate
Source: Chem Sci. 2021 Feb 11;12(12):4450–4. doi: 10.1039/d0sc04647g (PMC8179479; doi:10.1039/d0sc04647g)
Supplement: SC-012-D0SC04647G-s001 [file SC-012-D0SC04647G-s001.pdf]

Electronic Supplementary Material (ESI) for Chemical Science.
This journal is © The Royal Society of Chemistry 2021

Supporting information

Optimal water concentration for aqueous Li^+ intercalation in vanadyl phosphate

Dan Sun,[1] Masashi Okubo,[1,2] and Atsuo Yamada[1,2,*]

[1]Department of Chemical System Engineering, School of Engineering, The University of Tokyo, Hongo 7-3-1, Bunkyo-ku, Tokyo 113-8656, Japan

[2]Elements Strategy Initiative for Catalysts & Batteries (ESICB), Kyoto University, Nishikyo-ku, Kyoto 614-8245, Japan

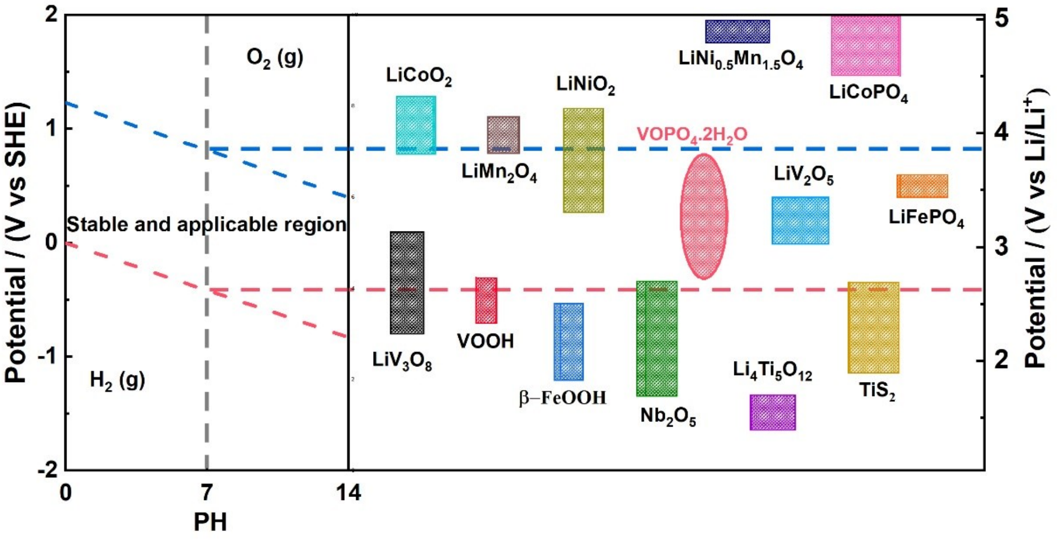

Figure S1. Pourbaix diagram of water and electrochemical stability window of lithium intercalation potentials for commonly used electrode materials in the aqueous battery system.

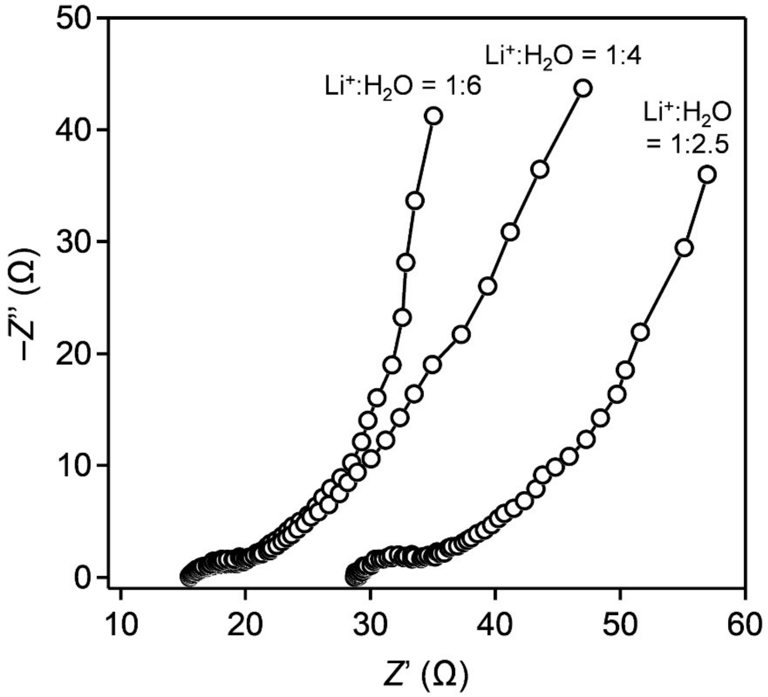

Figure S2. Nyquist plots for $VOPO_4 \cdot nH_2O$ electrodes at open circuit potential with aqueous Li^+ electrolytes at Li^+/H_2O ratio of 1:6 (LiTFSI/$6H_2O$), 1:4 (LiTFSI/$4H_2O$), and 1:2.5 (LiTFSI/$2.5H_2O$), where the electrode potentials are 1.01, 1.02, and 1.16 V vs. Ag/AgCl, respectively.

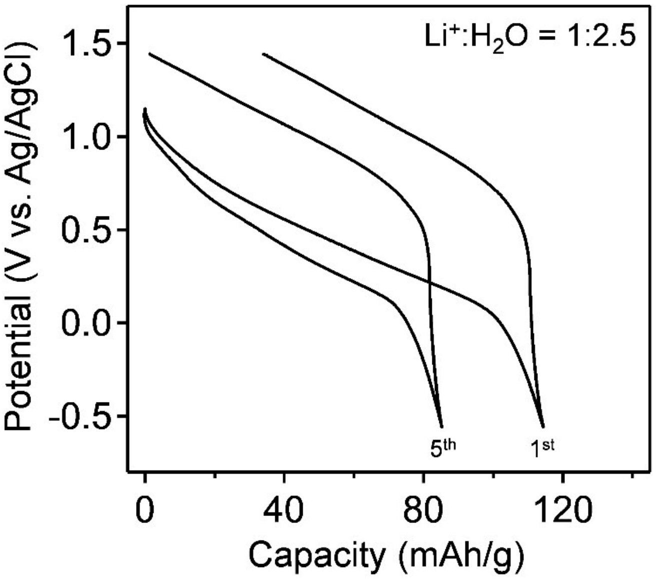

Figure S3. Charge/discharge curves of $VOPO_4 \cdot nH_2O$ electrodes with aqueous Li^+ electrolytes at a Li^+/H_2O ratio of 1:2.5 (LiTFSI/$2.5H_2O$). A specific current is 1 A/g.

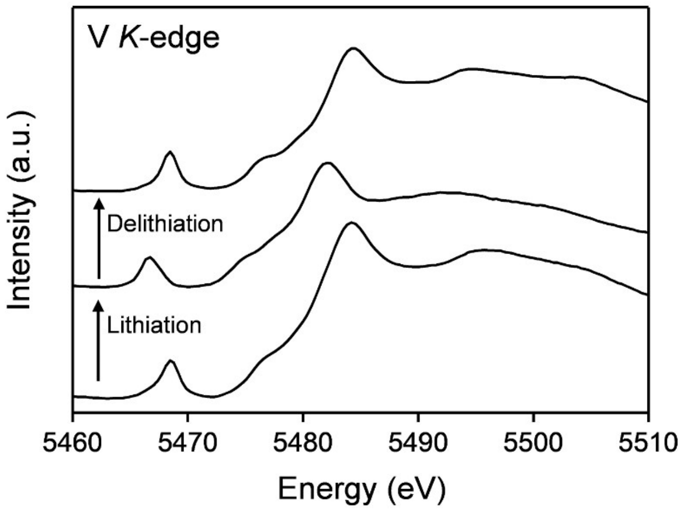

Figure S4. *Ex situ* XANES spectra for V *K*-edge of (de)lithiated $VOPO_4{\cdot}nH_2O$ in LiTFSI/$4H_2O$.

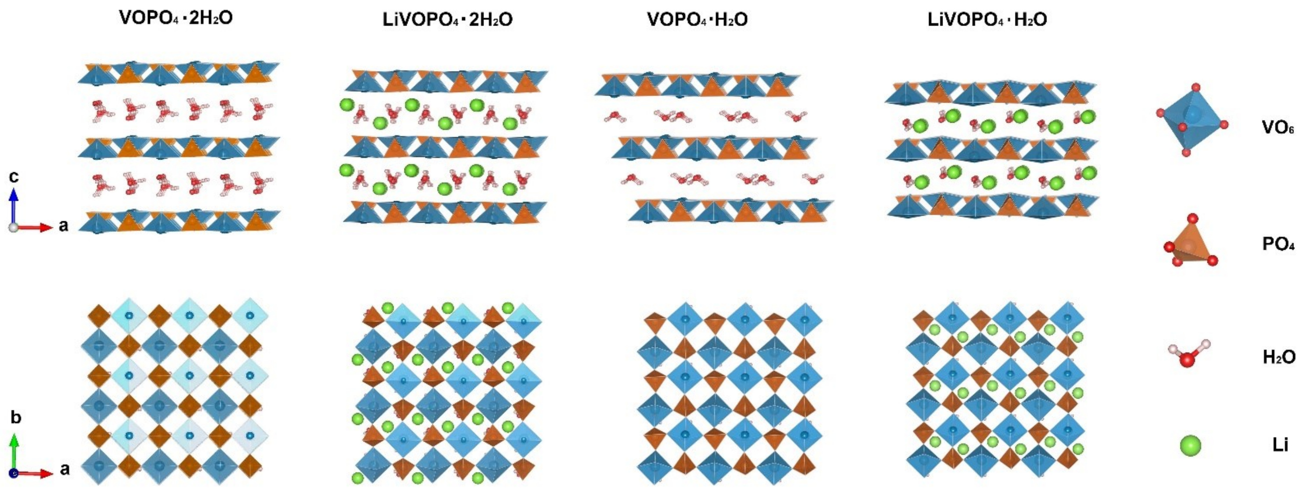

Figure S5. DFT+U+VDW optimized structures of $VOPO_4{\cdot}2H_2O$, $LiVOPO_4{\cdot}2H_2O$, $VOPO_4{\cdot}H_2O$ and $LiVOPO_4{\cdot}H_2O$, respectively

Table S1. DFT+U+VDW optimized lattice parameters for selected $Li_xVOPO_4 \cdot nH_2O$ (n = 0, 1, and 2) compound. The *c*-values represent the interlayer distance.

	a / Å	b / Å	c / Å	α / °	β / °	γ / °
$VOPO_4 \cdot 2H_2O$	6.26	6.21	7.25	91.7	90	90
$LiVOPO_4 \cdot 2H_2O$	6.32	6.42	6.56	90	75	90
$VOPO_4 \cdot H_2O$	6.25	6.24	6.16	105.3	90.6	90
$LiVOPO_4 \cdot H_2O$	6.39	6.45	5.53	105.6	91.5	89.8
